# Refractory macular hole surgery with amniotic membrane transplant functional assessment

**DOI:** 10.1186/s40942-025-00733-x

**Published:** 2025-10-14

**Authors:** Helena Proença, Marília Antunes, Joana Tavares Ferreira, Paula Magro, Mun Faria, Carlos Marques-Neves

**Affiliations:** 1 Faculdade de Medicina, Universidade de Lisboa, Lisboa, Portugal; 2 Vision Lab, Instituto de Saúde Ambiental, Faculdade de Medicina, Universidade de Lisboa, Lisboa, Portugal; 3 Serviço de Oftalmologia, ULSSanta Maria, Lisboa, Portugal; 4 CEAUL – Centro de Estatística e Aplicações, Faculdade de Ciências, Universidade de Lisboa, Lisboa, Portugal

**Keywords:** Amniotic membrane transplantation, Refractory macular hole, Functional outcomes, Multifocal electroretinography

## Abstract

**Purpose:**

To evaluate refractory macular hole (MH) surgery with amniotic membrane transplant functional outcomes.

**Methods:**

Prospective study over a five-year period. Nineteen patients with full-thickness macular hole (15 refractory MH and 4 chronic ≥ 8 years MH) were included. All patients underwent vitrectomy, cryopreserved amniotic membrane subretinal graft, tamponade and positioning. Functional outcomes were assessed using best-corrected visual acuity, M-CHARTS™ and mfERG (P1 and N1 waves amplitude and implicit time in five rings and ratios) preoperatively, at 3- and 12-months follow-up. Optical coherence tomography was also performed for structure-function analysis.

**Results:**

Mean follow-up was 45 months (range 15–68). Mean MH minimum linear diameter was 819 ± 242 μm and base diameter was 1387 ± 346 μm. After surgery, MH closed in all patients. Mean logarithm of the minimum angle of resolution (logMAR) visual acuity improved significantly from 1.70 ± 0.40 to 1.12 ± 0.39 (*p* < 0.001), mean first ring P1 amplitude, P1 implicit time, N1 amplitude and ratio 2 increased significantly (ß = 19.82, *p* = 0.029), (ß = 6.72, *p* = 0.033), (ß = 10.76, *p* = 0.007) and (ß = 0.836, *p* = 0.011), respectively. Foveal peak improved in 60% patients, one third presenting eccentric positioning.

**Conclusions:**

Visual acuity, metamorphopsia and mfERG improvements postoperatively suggest that amniotic membrane graft may be a useful tool to achieve refractory macular hole closure and enhanced functional outcomes.

## Introduction

Full-thickness macular hole (MH) is defined as a foveal lesion with interruption of all retinal layers from the internal limiting membrane (ILM) to the retinal pigment epithelium. With internal limiting membrane peeling, idiopathic macular hole closure rate is around 95–98% [1]. The inverted internal limiting membrane flap technique has improved the outcomes in large and myopic macular holes [2]. Still, refractory and chronic macular holes remain surgical challenges. Several techniques have been described with variable results: heavy silicone oil [3], macular detachment [4], autologous transplantation of the internal limiting membrane [5], autologous neurosensory retinal free flap [6], lens capsular flap transplantation [7], among other. In 2018 human amniotic (AM) membrane subretinal implant is implemented in recurrent macular holes and complex retinal detachments with encouraging results [8]. Revisional surgery for persistent idiopathic MH comprising ILM peeling enlargment and tamponade alone may be an option, especially for smaller refractory holes [9]. When there is no ILM left, specially for refractory macular holes larger than 680 micron higher closure rate is achieved with AM transplant compared to autologous ILM free flap transplantation [10]. Further evidence of the tecnique effectiveness comes from recent studies, meta-analysis and systematic review [11–13].

Most amniotic membrane transplant reports highlight the good anatomical outcomes and visual acuity improvements but further macular function assessment is frequently overlooked.

Multifocal electroretinography (mfERG), developed in 1992, allows an objective measurement of the electrophysiological responses of the central retina [14]. The mfERG responses are predominantly generated by the cone photoreceptors and its pathway, enabling topographic mapping of retinal function in the central 40–50° of the retina [15]. It has been investigated in macular function testing in multiple retinal diseases and is a useful tool to detect funtional changes after macular hole surgery [16].

This is a prospective study designed to address macular function testing in patients with refractory and large chronic macular hole.

## Methods

### Setting and patients

This is a prospective study including nineteen patients. The mean follow-up period was 45 months (range 15–68). The study design adhered to the tenets of the Declaration of Helsinki and was approved by the Ethics Committee of ULSSM/CAML. Written informed consent from all patients was obtained before the surgical procedure.

Best-corrected visual acuity (BCVA) was measured using the ETDRS Chart and expressed as the logarithm of the minimum angle of resolution (logMAR) for statistical analysis. Hand motion was considered as logMAR 2.3 and counting fingers at 1 m as logMAR 1.8. M-CHARTS™ (Inami Co., Tokyo, Japan) was used to quantify vertical and horizontal metamorphopsia.

### Optical coherence tomography

Retinal images were acquired using Spectralis Spectral Domain – Optical Coherence tomography (OCT) (Heidelberg Engineering, Heidelberg^®^, Germany) with posterior pole images centered on the fovea (61 acquisitions, 120 μm interval) and six-line radial scans. Preoperative OCT hole size – widest basal diameter and minimal linear diameter was determined.

### Multifocal electroretinography

RETIscan MultifocalERG (mfERG) (RETIport/Scan21 Gamma Plus²; Roland Consult) was used for mfERG recording. The recording procedures were the same as those described by the International Society for Clinical Electrophysiology of Vision and according to 2021 update [17]. The stimulus consisted of 61 hexagons that scale concentrically and covered the central 25 degrees of the fundus area. The viewing distance was 28.5 cm, which allowed a viewing angle of approximately 30 degrees. Each hexagon was modulated temporally between black (2 cd/m^2^) and white (200 cd/m^2^) - pattern reversal. The recording was performed with the DTL silver thread electrode, placed in the fornix of the lower eyelid after topical anesthesia, while the silver-chloride reference electrode was placed on the ipsilateral temple, and the ground electrode was positioned on the forehead. The recordings were performed before other testing to prevent retinal cells saturation. Subjects were light-adapted for at least 15 min and room lights maintained on during the recording. Pupils were dilated with tropicamide and phenylephrine hydrochloride. Refractive errors were corrected and added + 3.00 diopters. Clarity of the ocular media was assured. The examination was performed in each eye separately, while the other eye was occluded. During the recordings, patient fixation was monitored using an infrared camera incorporated in the device. The signal was amplified (100,000) and bandpass filtered (10–300 Hz). Three-dimensional topography represents the retinal response density (amplitude per retinal area, nV/deg^2^). The typical waveform of the basic mfERG response is a biphasic wave with an initial negative deflection, N1 wave, followed by a positive peak, the P1 wave. Implicit times (latencies) and the amplitude relative to their respective areas (nV/deg^2^) of N1 and P1 waves were measured using regional averages derived from 5 concentric rings. Three-dimensional topography represents the retinal response density (amplitude per retinal area, nV/deg^2^). The studied field contained 61 hexagons in 5 rings within a field diameter of 25 degrees, 12.5 degrees radially centered on the fovea and was analyzed with RETIscan software. For evaluation of the function at the region of amniotic membrane transplant, response densities in the first two rings were analyzed. The first ring corresponded to the foveal 5° area (0°- 2.5° viewing angle) and the second ring corresponded to the parafoveal region up to 16° (2.5°- 8° viewing angle). As 1° of visual angle is equal to 288 μm on the retina, without correction for shrinkage, the first ring tested the function approximately to 720 μm eccentricity (diameter 1440 μm) and the second ring from 720 to 2304 μm (diameter 4608 μm) [18]. Multifocal ERG was recorded preoperatively, at 3- and 12-months follow-up. Ring ratios, defined as the ratios of the central ring response density (R1) to the peripheral ones (R1/R2, R1/R3, R1/R4, R1/R5), presence or absence of foveal peak and peak eccentricity were also recorded for analysis.

### Surgical procedure

We performed 23-gauge three-port pars plana vitrectomy, cut the cryopreserved human amniotic membrane, introduced the flap through the trocar with forceps and placed the graft in the subretinal space with the epithelium, the non-sticky side, facing the vitreous cavity. Fluid air exchange and endotamponade with Sf6, C3F8 or silicone oil were performed. Patients were instructed to maintain 5-day face-down position. Surgeries were performed by experienced surgeons (HP, MF, CMN).

### Statistical analysis

Quantitative variables were described by mean ± standard deviation, median and range, categorical variables were described by absolute and relative frequencies (%). Pearson correlation was used to measure association between quantitative characteristics. Wilcoxon signed rank test was used to compare preoperative and postoperative BCVA. Student-t test for paired data was used to assess the significance of the mean change in the BCVA. Multiple linear regression was used to evaluate the relation between preoperative and postoperative BCVA, while controlling for the effect of possible confounders. Linear mixed effects models were used to assess the change in amplitude, latency and ratio of the amplitudes over time.

All statistical analyses were performed using R (R Core Team, Version 4.4.3) and RStudio (RStudio Core Team, Version 2024.12).

## Results

### Demographic and clinical data

The median patient age was 72.4 ± 8.9 years (range 50–88), seven male and twelve female patients, 13 White and 6 Black patients. Fifteen patients (78.9%) were pseudophakic with previous phacoemulsification and failed macular hole surgery. The other four phakic cases (21.0%) were chronic, long-standing MH (8–30 years) (Table 1).

**Table 1:** Demographic and OCT characteristics

Patient	Age (years)	Previous Treatment/Duration of Symptoms (years)	Axial Lenght (mm)	Baseline BCVA LogMAR (Snellen)	Final BCVA LogMAR (Snellen)	MH Linear Minimum Diameter (µm)	MH Base Diameter (µm)	Final Anatomical Success	Tamponade	Lens Status
1	66	Phaco, PPV + ILM peeling	-	1.8 (20/1260)	1 (20/200)	1171	1763	Closed	SF6	PC-IOL
2	64	Phaco, PPV + ILM peeling	24.23	1.8 (20/1260)	1.3 (20/400)	1528	2117	Closed	SF6	PC-IOL
3	74	Phaco, PPV + ILM peeling	22.53	1.8 (20/1260)	1.3 (20/400)	577	1498	Closed	SF6	PC-IOL
4	81	Phaco, PPV + ILM peeling	22.78	1.8 (20/1260)	1.3 (20/400)	730	1163	Closed	SF6	PC-IOL
5	78	Phaco, PPV + inverted ILM flap, PPV + autologous retinal transplant	23.73	2 (20/2000)	0.8 (20/125)	1119	1209	Closed	SO	PC-IOL
6	50	Phaco, PPV + ILM peeling	26.18	1.8 (20/1260)	0.8 (20/125)	742	1356	Closed	C3F8	PC-IOL
7	76	Phaco, PPV Retinal detachment, PPV subluxated IOL, PPV MH + ILM peeling	-	2.3 (20/4000)	2.3 (20/4000)	776	961	Closed	SF6	Iris sutured-IOL
8	84	> 15	22.58	1.8 (20/1260)	1.2 (20/320)	866	1347	Closed	SF6	Phakic
9	69	Phaco, PPV + ILM peeling	22.94	1.2 (20/320)	0.9 (20/160)	806	1529	Closed	SF6	PC-IOL
10	62	Phaco, PPV hemovitreous and MH + ILM peeling + anti-VEGF	22.34	1.1 (20/250)	0.8 (20/125)	637	1370	Closed	SF6	PC-IOL
11	84	> 18	21.80	1.1 (20/250)	0.8 (20/125)	720	1362	Closed	SF6	Phakic
12	76	Phaco, 2 PPV + inverted ILM flap	24.15	2 (20/2000)	1.9 (20/1600)	524	1218	Closed	SF6	PC-IOL
13	71	Radial Keratotomy + Phaco + anti-VEGF + Retinal Detachment	31.17	2.3 (20/4000)	1.1 (20/250)	559	2242	Closed	C3F8	PC-IOL
14	70	> 8	23.72	1.3 (20/400)	0.8 (20/125)	939	1320	Closed	SF6	Phakic
15	88	> 30	22.42	2 (20/2000)	1 (20/200)	769	1503	Closed	SF6	Phakic
16	73	Phaco, PPV + ILM peeling	21.92	1 (20/200)	0.9 (20/160)	763	947	Closed	SF6	PC-IOL
17	74	Phaco, PPV + inverted ILM flap, VPP + Epiretinal Amniotic Membrane Transplant	22.45	2 (20/2000)	1.9 (20/1600)	902	1054	Closed	C3F8	PC-IOL
18	66	Penetrating keratoplasty, Phaco, PPV + inverted ILM flap + PRGF-Endoret^®^	33.12	1.8 (20/1260)	1 (20/200)	629	1316	Closed	SF6	PC-IOL
19	70	Phaco, PPV + ILM peeling	-	1.3 (20/400)	1 (20/200)	801	1079	Closed	SF6	PC-IOL

anti-VEGF = anti-vascular endothelial growth factor; BCVA = best-corrected visual acuity; ILM = internal limiting membrane; IOL = intraocular lens; MH = macular hole; OCT = optical coherence tomography; PC-IOL = posterior chamber intraocular lens; PPV = pars plana vitrectomy

PRGF- Endoret^®^ = plasma rich in growth factors-Endoret^®^

Macular hole closure was achieved in all patients. There were no complications except one ocular hypertension case controlled with topical therapy and one transient vitreous cavity hemorrhage.

Visual acuity improved between preoperative and last observation in all patients except for one that maintained preoperative visual acuity (Wilcoxon test for paired data, *p* < 0.001). The mean preoperative logMar visual acuity (Snellen units) was 1.70 ± 0.40 (20/1000), median 1.80 which improved significantly to logMAR (Snellen units) 1.12 ± 0.39 (20/250) median 1.00: the mean change in logMar visual acuity was −0.58 ± 0.38 (t-test for paired data, *p* < 0.001, 95% CI for mean change (−0.76, −0.40). A linear regression model was used to rule out the possible confounding effect of lens status in the relation between postoperative and preoperative BCVA. Results showed no significant effect (ß_Lens status_ = 0.11, *p* = 0.568).

Reliable pre and post-surgery metamorphopsia scores evaluations were obtained for 5 patients. The vertical metamorphopsia score improved in all cases (min = 0.4, median = 0.5, max = 2.4). The horizontal metamorphopsia score improved in 4 of the 5 cases (min = 0.4. median = 1.1, max = 2.4). One patient had the horizontal metamorphopsia score worsened by 0.1. The changes in vertical metamorphopsia score were borderline statistically significant (Wilcoxon test, *p* = 0.058) and no significant change was found in horizontal metamorphopsia scores from preoperative evaluation to postoperative evaluation.

### OCT

The mean preoperative macular hole size was 819 ± 242 μm (median 763 μm) at the minimum linear diameter and 1387 ± 346 μm (median 1347 μm) at the base diameter. Centered and matched OCT scans demonstrated hole closure in all patients, including cases already refractory to previous rescue surgeries usually recommended for difficult cases as autologous neurosensory free-flap retinal transplant technique (patient 5), epimacular amniotic membrane transplant (patient 17) or plasma rich in growth factor (ENDORET^®^) (patient 18).

**Fig. 1:**
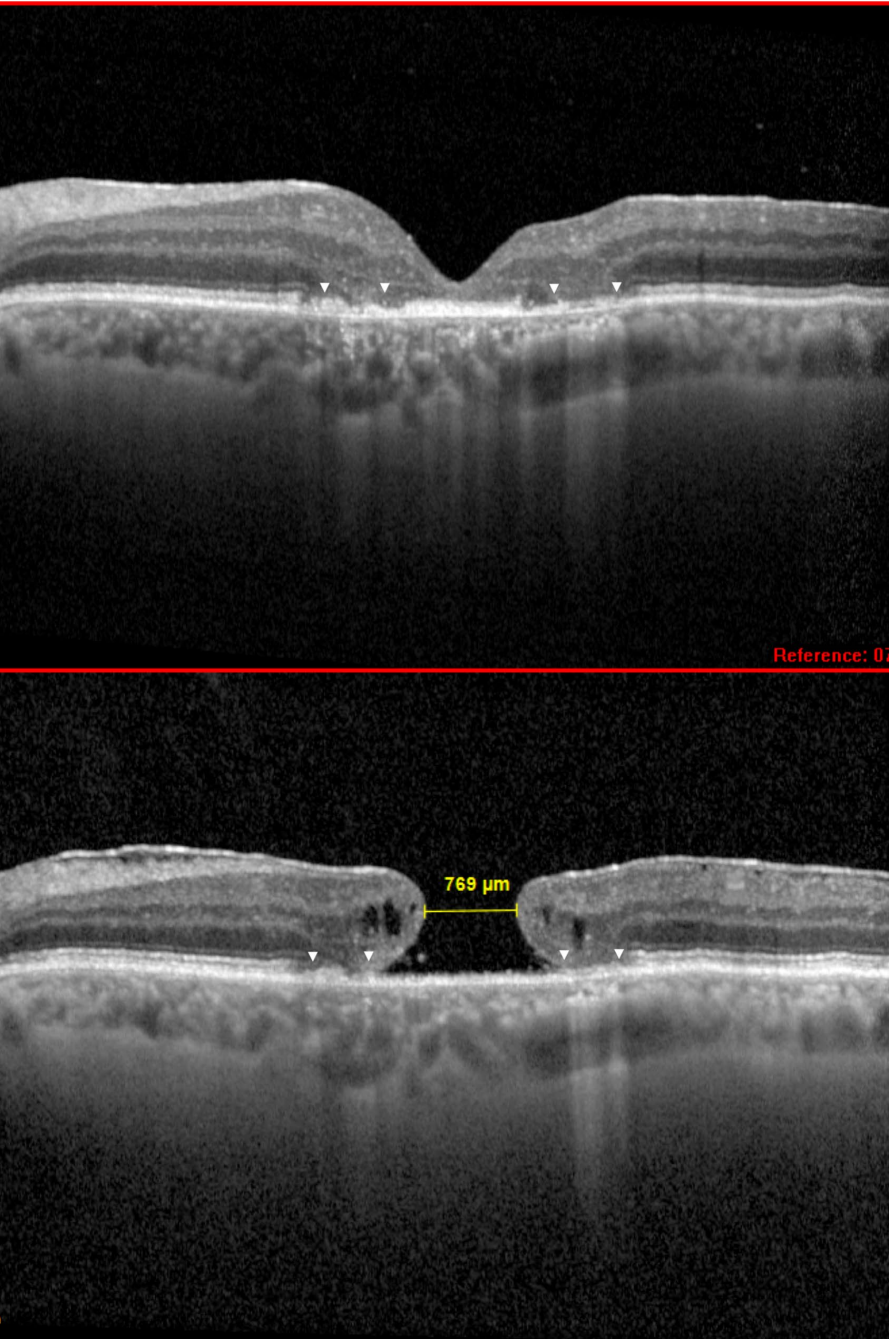
Centered and matched optical coherence tomography (OCT) scans preoperatively and postoperatively: Patient 15. A flat macular hole documented for 30 years closed after subretinal amniotic membrane transplant. Postoperative perifoveal external layers atrophy (top, arrowheads) were already present preoperatively (bottom, arrowheads)

At the early postoperative period, we observed inner retinal layers rearrangement and ingrowth plunging into the hole (Fig. 1) and/or a thin epiretinal layer spanning the central amniotic membrane graft over the hole. Minimum linear diameter and MH base diameter did not correlate significantly with final BCVA, (cor = −0.134, *p* = 0.59) and (cor = −0.143, *p* = 0.56), respectively.

### ERG

After surgery, there was a statistically significant increase in mean P1 amplitude in the first ring at 3-months follow-up (ß = 19.82, *p* = 0.029), that covered the region of the graft. No significant change was found between 3- and 12-months follow-up (*p* = 0.185) (Table 2).

**Table 2:** Multifocal ERG results before, at 3-months and 12-months after surgery

Parameter	Preop	3-m Postop	12-m Postop	*p* value	
P1 amplitude (nV/deg^2^)				Preop to 3-m Postop	3- to 12-m Postop
Ring 1	37.43±14.84	57.07±20.74	47.44±20.82	**0.029**	0.185
Ring 2	28.20±9.32	27.66±7.89	32.14±12.76	0.420	0.178
Ring 3	21.74±7.77	24.93±8.39	24.99± 9.03	0.485	0.938
Ring 4	15.12±6.11	16.98±6.45	16.49±6.21	0.680	0.936
Ring 5	10.72±4.87	14.22±4.29	11.61±4.90	0.190	0.468
P1 latency (ms)					
Ring 1	36.29±6.76	43.01±4.59	38.43±5.67	**0.033**	0.114
Ring 2	39.23±5.83	39.07±3.32	39.28±2.38	0.844	0.739
Ring 3	36.27± 3.60	36.54±1.94	39.24±2.88	0.869	0.068
Ring 4	37.97±1.91	38.26±1.37	38.39±2.69	0.674	0.725
Ring 5	37.71± 2.16	38.11±1.91	39.30±3.09	0.549	0.736
Ratios P1					
R1/Rx					
Ring 1	1	1	1	-	-
Ring 2	1.46±0.71	2.13±0.72	1.69±1.01	**0.011**	**0.027**
Ring 3	3.59±6.57	2.40±0.68	2.06±1.01	0.543	0.850
Ring 4	3.88±4.42	3.50±0.90	3.19±1.71	0.890	0.668
Ring 5	6.56±8.99	4.04±1.00	4.44±2.30	0.469	0.850
N1 amplitude (nV/deg^2^)					
Ring 1	15.32±7.50	25.63±9.60	13.26±8.08	**0.007**	**0.002**
Ring 2	10.02±5.42	5.74±1.26	8.46±5.30	0.055	0.221
Ring 3	7.88±3.54	6.80±2.13	7.58±2.70	0.440	0.564
Ring 4	5.52±2.51	4.12±1.88	4.86±2.25	0.133	0.408
Ring 5	3.66±2.42	3.94±1.33	3.59±1.42	0.752	0.677
N1 latency (ms)					
Ring 1	20.76±5.98	20.73±4.62	20.21±5.10	0.917	0.678
Ring 2	17.84±3.56	21.57±2.38	19.60±2.20	**0.014**	0.139
Ring 3	16.31±4.08	19.33±1.77	19.03±3.65	0.076	0.718
Ring 4	17.18±3.42	20.19±2.04	19.49±2.11	**0.027**	0.560
Ring 5	18.02±1.59	19.47±1.92	20.04±2.48	0.152	0.599
Ratios N1					
R1/Rx					
Ring 1	1	1	1	-	-
Ring 2	1.96±1.46	4.70±2.44	2.83±3.28	**0.018**	**0.073**
Ring 3	4.25±8.45	3.96±1.17	1.86±1.12	0.933	0.358
Ring 4	3.59±3.15	6.99±3.51	3.41±2.70	**< 0.001**	**< 0.001**
Ring 5	13.08±22.29	7.03±3.52	3.94±2.22	0.402	0.530

m = months; Preop = preoperatively; Postop = postoperatively

Amplitude of P1 and N1 are expressed in nanovolts (nV) per degree squared (deg^2^) and implicit time (latency) in milliseconds (ms). All values are expressed as mean ± standard deviation. Bold values indicate statistically significant p values

Postoperatively, we found a statistically significant increase in mean P1 implicit time in the first ring at 3-months (ß = 6.72, *p* = 0.033). No significant change was found between 3- and 12-months (*p* = 0.114).

There was a statistically significant improvement in mean N1 amplitude in the first ring at 3-months follow-up (ß = 10.76 *p* = 0.007), with a return to baseline values at the latest postop follow-up.

Postoperatively, we also found an increase in N1 implicit time, although generally not statistically significant, except for ring 2 (ß = 3.74, *p* = 0.014) and ring 4 (ß = 2.97, *p* = 0.027) at 3-months follow-up.

In rings 2,3,4 and 5 amplitudes and implicit times of P1 and N1 did not change significantly during all follow-up evaluations.

After surgery, there was a statistically significant increase in Ratio 2 at 3-months observation (ß = 0.836, *p* = 0.011). No significant change was found in peripheral rings ratios during follow-up.

We found no correlation between mfERG amplitudes, implicit times or Ratios and BCVA or BCVA gain preoperatively or at 3-months postoperatively.

**Fig. 2:**
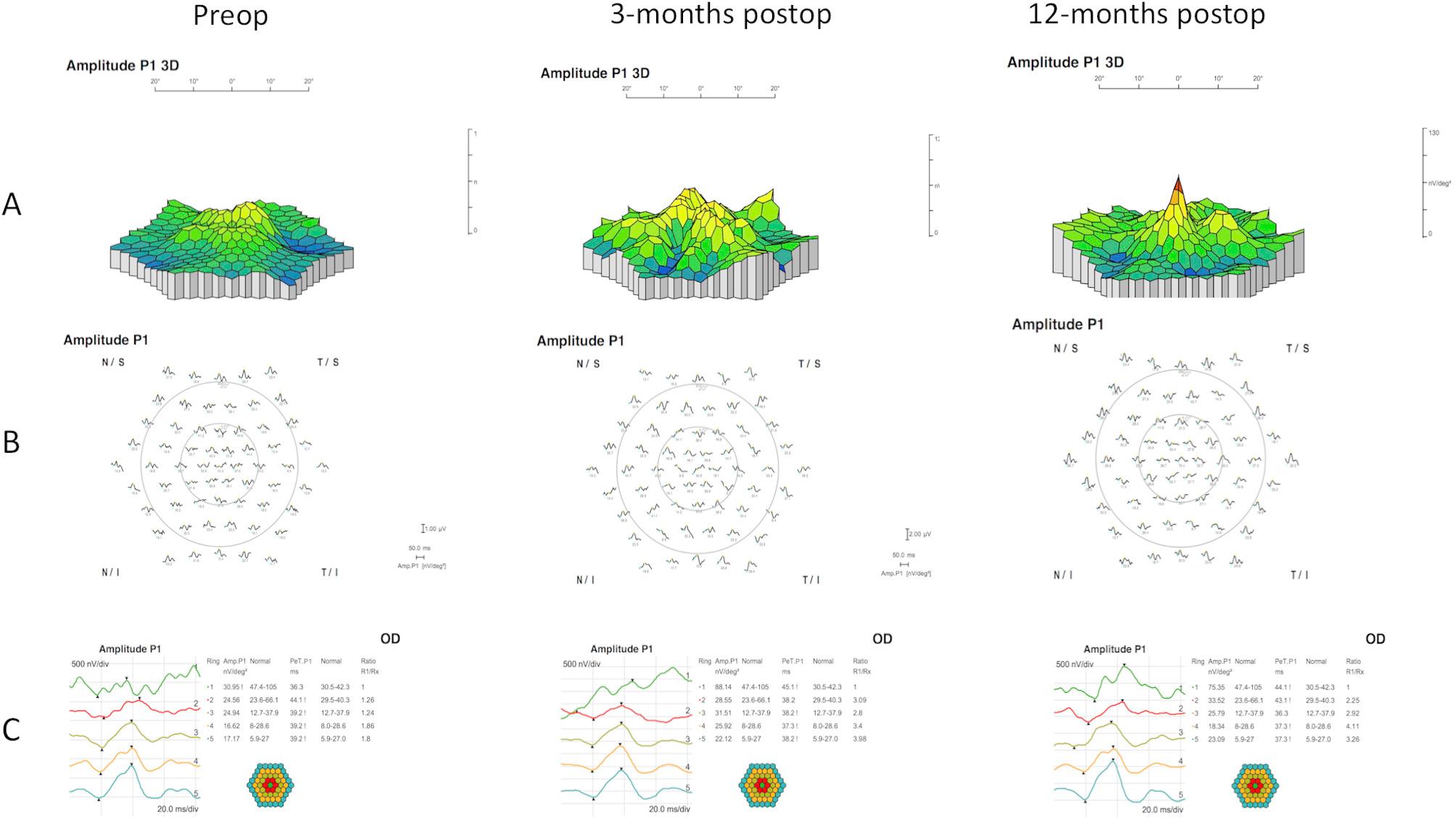
Multifocal ERG responses: preoperatively, 3- and 12-months postoperatively of the case no. 16. **A**: Three-dimensional color plot topography of amplitude P1. **B**: Trace arrays: amplitude of P1 in nV/deg^2^ in topographic display around the fovea. **C**: P1 wave ring analysis: Amplitude, latency and Ratio. Three-months after surgery, the responses showed increased P1 wave amplitude in the 1st and 2nd ring (corresponding to 0–2.5° foveolar and 2.5–8° parafoveolar regions) with abnormal wave morphology in the 1st ring but near normal in the 2nd ring (B and C, middle) and stable 12-months after surgery (B and C, right). Foveal peak outline was improved at 12-months postoperatively (A, right). Abbreviations and Acronyms: BCVA = best-corrected visual acuity; ILM = internal limiting membrane; logMAR = logarithm of the minimum angle of resolution; mfERG = multifocal electroretinogram MH = macular hole; OCT = optical coherence tomography

Foveal peak outline was identifiable in 60% patients after surgery (Fig. 2), a third of them presenting an eccentric positioning.

Despite postoperative improvements there was a reduced response with abnormal waveform morphology in the central foveolar zone.

When comparing BCVA vs. Ratio we found that Ratio 2 had no association with logMAR BCVA. Ratio 3 and 4 had a 10% significant association: higher logMAR associates with higher Ratio 3 (*p* = 0.083) and higher Ratio 4 (*p* = 0.06). Ratio 5 had a 5% significant association: higher logMAR associates with higher Ratio 5 (*p* = 0.048). Ratio vs. logMAR BCVA associations were valid before or after surgery. At any evaluation moment, for each increase point in Ratio there was an increase in logMAR of 0.029, meaning that patients with higher ratio (e.g. best at ring 5 than ring 1) have worst visual acuities. These results suggest the larger the difference between external and central ring amplitudes (meaning that the central amplitude is reduced compared to external), the worse the visual acuity. These results agree to our mfERG results validation.

## Discussion

Subretinal amniotic membrane transplantation is successful at closing large refractory macular holes. The procedure is not technically demanding, provides high anatomical success rate and has very low complication rate even in long-term follow-up [19–20]. In the present study hole closure was achieved in all patients including cases of failed rescue techniques recommended for refractory macular holes as plasma rich in growth factor-ENDORET^®^, epimacular amniotic membrane transplant and autologous neurosensory free-flap retinal transplant techniques.

Encouraged by preliminary results this prospective study was designed to study the functional outcomes of amniotic membrane transplanted patients for refractory macular hole by mfERG [21].

The mfERG is a non-invasive technique for assessing the local ERG from different regions of the posterior retina. In the present report we studied the N1 wave, which originates from cone cells, hyperpolarizing bipolar cells and Müller cells and the P1 wave, which derives from depolarizing bipolar cells, hyperpolarizing bipolar cells and Müller cells [22].

We found a statistically significant increase in mean N1 e P1 amplitude at 3-months postoperatively (*p* = 0.029), in the first ring. As the first ring tests approximately the central 1440 μm of the macula and our preoperative bare retinal pigment epithelium area, correspondent to preoperative macular hole mean base diameter, was 1387 ± 346 μm, these results reflect some functional recovery of the central function of the macula covered and bordered by the graft. Still, we found an abnormal waveform morphology and subnormal values in the central foveolar zone indicating most probably outer retinal layers dysfunction and cell loss, although a contribution of fixation instability in patients with poor visual acuity leading to reduced amplitudes cannot be excluded [23].

Even after idiopathic macular hole surgery, longstanding alteration of cone function reflected by mfERG has been verified 18-months after surgery [24]. Perifoveal functional impairment extending beyond the macular hole was also previously demonstrated in mfERG studies with amplitudes reduced in a 7.8° diameter central area [25]. Our series included mostly refractory holes, with multiple surgeries where much cell damage had already occurred. Therefore, the increased amplitudes after surgery suggest a functional benefit from MH closure with the amniotic membrane transplant.

The P1 wave implicit time was significantly prolonged in the first ring 3-months postoperatively. Prolonged implicit time has been demonstrated in diabetic macular edema after photocoagulation and may reflect functional impairment of the retina due to ischemia, hypoxia or local metabolic changes [26]. In macular hole mfERG reports implicit times are reported by only a few authors. A delayed implicit time was observed in idiopathic macular holes undergoing standard ILM peeling [24]. In the inverted internal limiting membrane flap technique, the implicit time of P1 was significantly longer in operated eyes than in the fellow eyes and this was attributed to ILM peeling trauma, as the ILM is the basement membrane of Müller cells [27]. In the present study, most (79%) of our cases were refractory holes with no ILM left and therefore no additional mfERG changes related to ILM peeling were expected, whether due to staining or mechanical striping of Müller cells end-feet [28–29].

We found a statistically significant increase in Ratio 2 after surgery reflecting the increase in the first ring amplitude as no relevant change was seen in the second ring amplitude. This increase agrees with previous studies with the ILM peeling technique and the inverted ILM flap technique [30], suggesting that macular hole closure with the amniotic transplant technique also leads to improved functional outcomes. As expected, no significant change was found in peripheral rings ratios during follow-up.

There was no direct relation between visual acuity and mfERG parameters, even in the most central rings 1 and 2, in accordance with visual acuity being dependent on the function of a very few central photoreceptors whereas the mfERG responses reflecting an averaged response over a retinal area.

In the present study the postoperative functional improvement, expressed by the increase in P1 and N1 waves amplitude, seems to occur despite disruption of photoreceptor layer seen on several OCT. Also, on OCT, we observed postoperative improvement of the inner retinal layers, even in patients whose photoreceptor layer remained disrupted. Overall, our results reflect outer retinal layers partial functional improvement, related primarily to cone-photoreceptors and bipolar cells, although the delayed N1 and P1 waves still denote cell disfunction.

The follow-up analysis showed that functional improvement matched structural recovery, occurring both in the early postoperative period. Afterwards, they remained stable during long-term follow-up. We hypothesize that no further outer retinal layers remodeling has occurred after hole closure, which may explain the modest final visual acuities achieved in these refractory macular holes as compared to idiopathic.

The effect of IOL implantation on mfERG has been previously studied: patients with an implanted IOL presented lower amplitudes [31]. Nevertheless, our population was already largely pseudophakic, so this variable did not interfere with our results.

Qualitative evaluation of the foveal peak outline in the three-dimensional color plot topography of amplitude P1 demonstrated an identifiable foveal peak, although attenuated, in 60% patients after surgery.

An eccentric positioning of the foveal peak to the parafovea was present in a third of these patients which may also contribute to functional improvement. This finding agrees with eccentric fixation shifting/learning in about 20–30% of patients after successful idiopathic macular hole surgery, reported in visual acuity measurements and confirmed in microperimetry testing [32].

Our results agree with mfERG improvements in autologous retinal transplantation. Additionally, subretinal rather than epiretinal graft placement led to better structural recovery [33].

Metamorphopsia is not usually evaluated in refractory macular hole surgery. Patients low visual acuities restrict the number of reliable M-CHARTS^™^ tests, precluding further statistical analysis. Nevertheless, we found a metamorphopsia score improvement after surgery in accordance with idiopathic macular holes studies [34–35]. Besides being refractory, macular holes included in this series are very large and MH diameter seems to be associated with metamorphopsia scores [35]. As described for idiopathic macular holes, we also found vertical metamorphopsia scores consistently better than horizontal metamorphopsia scores [34].

Regarding demographics, our race sample profile comprised 68% White patients and 32% Black patients which is largely disproportional to the country population. Since 79% of our cases were refractory MH, the high prevalence of Black individuals in our sample comes in line with the recently published twofold greater risk of failed full thickness macular hole surgery in these patients, for still uncertain reasons [36].

The present study has some limitations. This is a non-comparative study that lacks a control group as denying surgery to some patients would be unethical. Fellow eyes of patients with macular hole could not be used as controls as they have lower central amplitude and lower ring ratios [37]. Normative data in mfERG may vary with each equipment and population-specific factors such as age, ethnicity, pupil size, axial length, and diurnal variation. To obviate this limitation, intraindividual comparisons were obtained.

This study adds insight to previous reports of macular hole surgery with the subretinal amniotic membrane technique functional outcomes.

To the authors’ knowledge, this is the first study of macular function including multifocal electroretinogram assessment in macular hole surgery with amniotic membrane transplant.

In summary, the subretinal amniotic membrane transplant surgery in refractory macular holes is a very effective, low complication rate technique, that leads to visual acuity, metamorphopsia and electrophysiological functional improvements.

## Data Availability

No datasets were generated or analysed during the current study.
